# The use of life stories and its influence on persons with dementia, their relatives and staff – a systematic mixed studies review

**DOI:** 10.1186/s12912-017-0223-5

**Published:** 2017-06-02

**Authors:** Vigdis Abrahamsen Grøndahl, Mona Persenius, Carina Bååth, Ann Karin Helgesen

**Affiliations:** 1 Faculty of Health- and Social Studies, Østfold University College, 1757 Halden, Norway; 2 Faculty of Health, Science, and Technology, Department of Health Sciences, Karlstad University, Karlstad, Sweden

**Keywords:** Dementia, Life stories, Systematic mixed studies review, Nursing home

## Abstract

**Background:**

Dementia is an important predictor of nursing home admissions. Due to progressive dementia symptoms, over time it becomes difficult for persons with dementia to communicate their wishes and participate in decisions concerning their everyday lives. Their well-being, sense of dignity, integrity and personhood are at risk. The persons’ life stories have been highlighted as particularly important in dementia care and are referred to as seeing the person beyond the dementia. The aim of this study was to explore and describe the use of life stories and its influence on persons with dementia living in nursing homes, their relatives and staff.

**Methods:**

A systematic mixed studies review was conducted. The literature searches were performed in the following databases: CINAHL, PubMed and PsycINFO and the Cochrane library, as well as by hand searching references in the studies included. An updated search was performed eight months after the first search. Data was synthesised inspired by integrative analysis.

**Results:**

Three studies using quantitative design and two studies (presented in three papers) using qualitative design representing research from 2006 to 2015 were included in the review. Life stories generally had a positive influence on the persons with dementia, their relatives, and staff. The use of life stories might contribute to ‘Maintenance of the person with dementia as a whole person rather than a demented patient’. On the other hand, enabling persons with dementia to tell their own story could be a challenge. For the staff it could be challenging when sensitive information emerged uninvited. Involving relatives could also be difficult as to whose story were uncovered.

**Conclusions:**

The use of person’s life story might be of significance, but there is not enough evidence to make any statement about its importance as the research is scarce. Studies, including randomised controlled trials, are needed to measure the impact of life story work on the physiological and psychological aspects of persons with dementia, and also how it influences their relatives and staff.

## Background

Due to a greater prevalence of older people in society as a result of demographic changes, the incidence of persons with dementia has increased internationally [1, 2]. It is estimated that there will be 48.1 million persons with dementia globally by 2020. This is expected to reach 90.3 million by 2040 [3]. Persons with dementia need care and support in many areas of their lives [2] and dementia is an important predictor of nursing home admissions [4, 5].

Dementia is a collective term for several diseases that permanently and progressively reduce cognitive functions. Alzheimer’s disease is the most common cause of dementia, followed by vascular dementia, Lewy Body dementia and frontotemporal dementia [3, 6, 7]. As there is no medical curative treatment of dementia [2, 7], specific nursing care that positively affects the quality of life of the person is of the utmost importance [8, 9]. Kitwood’s work has been very influential in challenging the standard biomedical paradigm and offering an alternative for understanding dementia [8, 10, 11]. His conceptualisation of dementia highlighted the dialectic interplay between neuropathology and the social-psychological context of the individual, and contributed to the development of person-centred care within dementia care [11–13]. Since Kitwood’s work, various descriptions of person-centred care have been developed [14, 15]. Other related concepts such as relationship-centred care [16], senses framework [17], and dementia care nursing [18] are also presented. These descriptions and concepts have much in common by supporting the person’s rights, values and beliefs, and involving the person with dementia in decision making [8, 11, 14–16, 18].

It has been argued that it is the day-to-day decisions that are really omnipresent for persons with dementia [19, 20] as the possibility of participating in decisions concerning their everyday life will most likely impact the person’s well-being and sense of dignity, integrity, and personhood [11, 20–22]. However, due to progressive dementia symptoms such as increased cognitive and physical impairment, over time it will become more difficult for persons with dementia to express themselves, make choices, communicate their wishes and understand their present circumstances [23, 24].

When this happens, relatives may be entitled, according to political documents, legislation and nurses’ ethical codes [25–29], to participate, together with the person with dementia, in order to take care of the his/her interests. A recent dissertation [20, 30] has highlighted that relatives could be a link to good dementia care because by knowing the person’s life story and preferences they could share important information with nursing home staff. In a survey, the majority (98.7%) of relatives stated that they had good knowledge of the person’s habits and preferences. However, about 30% of relatives had not been asked to provide written information about their near ones, which shows that there is room for improvement in this issue [31]. Life story work that involves recording aspects of a person’s past and present life, and then using this information to benefit the person in his/her present situation [32], has been used in many countries within a range of health and social care settings [33–37].

Life stories are also used as a part of reminiscence work in which the person’s life reviews, often supported by photographs, artefacts and music, were actively used therapeutically [38, 39]. A review of intervention studies that focused on the use of life stories in the care of persons with dementia, found positive changes in the integration of self and that the quality of life was enhanced [38]. Life story works have been highlighted as particularly important in dementia care [40] and are referred to as seeing the person beyond the dementia [41].

Since no recent reviews were found that focused on how the life stories of persons with dementia influence persons with dementia, their relatives and staff, this study was carried out.

### Aim

The aim of this study was to explore and describe the use of life stories and its influence on persons with dementia living in nursing homes, their relatives and staff.

## Methods

A systematic mixed studies review with an integrated design was undertaken to integrate and synthesise results from quantitative, qualitative and mixed methods studies [42]. The design was chosen to explore and describe the use of life stories’ influence on the person with dementia, the relatives and staff. The research group strived to use the methodological guidance of the Cochrane Collaboration [43] in the search and also to structure the review together with the guidelines from “Preferred Reporting Items for Systematic Reviews and Meta-Analyses” (PRISMA) [44].

### Eligibility criteria

Eligibility criteria were based on the aim of the review and were determined before the literature search started. The aim was explorative and descriptive, and the inclusion criteria reflect this.

#### Participants and setting

Studies comprising the use of life stories for persons with dementia living in nursing homes were included. Studies concerning the use of life stories for people living at home who received home care were excluded. Furthermore, studies focusing on life stories regarding people with mental health problems were excluded to ensure that the focus remained on persons with dementia.

#### Type of studies

Peer-reviewed papers using qualitative, quantitative or mixed methods, and presenting research in English, Norwegian, Swedish or Danish, were included. There were no limits regarding date of publication beyond the coverage of the databases themselves. Reviews and books were excluded from the review.

#### Outcome

Studies were included when the outcome focused on the consequences of the use of life stories for the person with dementia, relatives and/or staff.

### Identification of relevant literature

#### Information sources

The literature searches were performed in three electronic databases: CINAHL via EBSCO, PubMed via NCBI and PsycINFO via OVID, as well as the Cochrane Library, 26 January 2015. An updated search was performed on 1 September 2015. ‘Dementia’ covering Dementia, Frontotemporal Dementia, Dementia Vascular, Dementia Multi-Infarct, Levy body Disease, Dementia Senile, Dementia Presenile, was used as a key search term in combination with the Boolean operator ‘AND’ with the search terms ‘biographical approach’ , ‘diary’ , ‘narratives’ , ‘life story’ or ‘life histories’ , and used as appropriate for the database. The full electronic search strategy for Pubmed is shown to exemplify the search: ((dementia [MeSH Major Topic] AND biographical approach), (dementia [MeSH Major Topic] AND diary), (dementia [MeSH Major Topic] AND narratives), (dementia [MeSH Major Topic] AND life story), (dementia [MeSH Major Topic] AND life history)). The references in the selected studies were scrutinised for further studies by looking for the key search phrases in the titles.

### Study selection

**Fig. 1:**
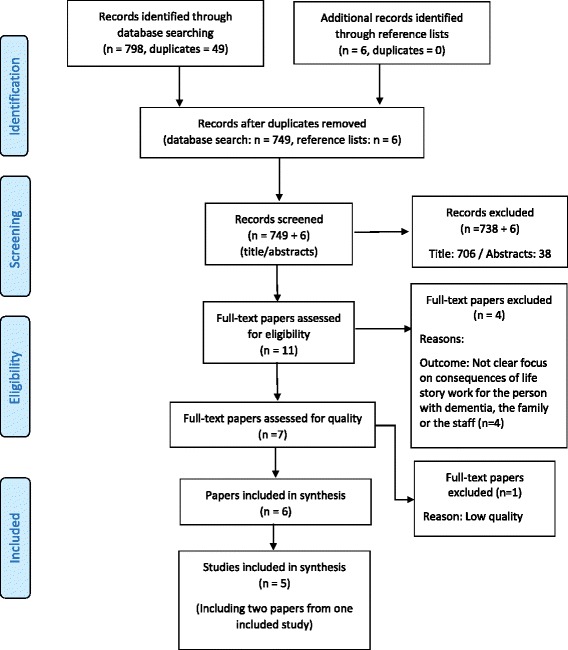
Flow diagram of the selection process. Source: modified version of flow diagram as reported by Moher et al. [44]

A modified flow diagram (Fig. 1) shows the procedure for the identification and selection process. The electronic database searches revealed 798 papers, with a further six papers identified through the reference lists. Duplicates were removed which resulted in 749 papers. All four authors independently read the titles. All titles were assessed according to their relevance to ensure that the eligibility criteria and the aim of the study were met and screened on a scale from 1 (relevant), 2 (maybe relevant) to 3 (not relevant). There were 706 papers with a score of 3 which were excluded. The papers with scores 1 and 2 on the title (49 papers) were divided into two equal parts and two authors read one half of the abstracts, the other two authors read the other half and gave each abstract a score from 1 to 3. Abstracts with score 1 or 2 were then read by all four authors, and the scores were subsequently discussed until consensus was reached. As a result, 38 papers were excluded, leaving 11 papers. The 11 papers were assessed in full text by all four authors independently to ensure that the inclusion criteria were met. Four papers were excluded, leaving seven papers to be assessed for quality.

### Appraisal and data extraction

The quality of each paper included in the review was assessed by two authors independently, using the Critical Appraisal Skills Programme Tools (CASP) for qualitative, quantitative and RCT studies [45], modified by Nordström and Wilde-Larsson [46, 47]. A scoring system was used to rate the studies as low, medium or high quality. Papers with low quality scores were then reassessed by the two other authors. Any differences on quality ratings were discussed in the research group until agreement was reached. One study was excluded based on low quality. In total six papers were included in the synthesis. Information regarding aim, study design, research type, sample, intervention, outcomes and key results were extracted from the studies. See Table 1 for characteristics of included studies and quality assessment.

**Table 1:** Characteristics of included studies and quality assessment

Authors/Country	Title	Aim	Design	Research type	Sample, including participants’ characteristics	Intervention	Outcome	Summary of relevant findings	Quality assessment
Buron 2010 [53] USA	Life history collages. Effects on nursing home staff caring for residents with dementia.	To compare changes in nursing staff knowledge of individual residents and their perceptions of knowing the person, staff- to- resident, communication, and staff –to-staff communication before and after exposure to life history collages.	A pilot study. A pretest-posttest, quasi-experimental design.	Quantitative	A convenience sample of five residents with dementia (3 intervention, 2 control) and 36 nursing staff members (18 intervention, 18 control), resident in two nursing homes. Person with dementia: Age: 63–95 years Gender: 1 woman and 4 men Length of stay: 10–11 months MMSE score: 1–11 Staff: Age: 19–62 years Gender: 30 women and 6 men Profession: 1 RN and 35 other Length of employment: 0- ≥ 10 years	Interviews with each resident’s surrogate were conducted and tangible (e.g., photos) and intangible (e.g., verbal description) information about each resident in three areas were collected: family, job/careers and likes/dislikes/interests. A graphic design artist used the information to create the essence of each resident’s participant’s life in the form of a life history collage that were placed on the residents’ room walls for a period of 4 weeks.	Nursing staff knowledge - author developed questionnaire Nursing staff perceptions of individualized care - IC-KNOW - IC-COMMUNICATION-SR - IC-COMMUNICATION-SS	The intervention group members’ knowledge of residents’ family, job/careers, and likes/dislikes/interests improved significantly at posttest and at 3 weeks post intervention. The perceptions of individualized care/person centered care practices did not improve significantly.	Medium
Haight, Gibson, Michel 2006 [50] Northern Ireland	The Northern Ireland life review/life storybook project for people with dementia.	To test the effectiveness of a structured life review/life storybook process.	A controlled pilot study. Pre- and posttest.	Quantitative	A convenience sample of 30 older people (15 intervention, 15 control) who had mild to moderate dementia, resident in six assisted-living facilities. Person with dementia: Age: 60–99 years Gender: 25 women and 6 men MMSE score: mean 17.84 at pretest	A series of semistructured interviews guided by the tool The Life Review and Experiencing Form (LREF) was conducted during 8 weeks. A life storybook was constructed by care staff and the participants in dyads. The life storybook was an illustrated loose-leaf binder with photographs and explanatory captions by using the elderly person’s own words.	The participants - cognition (MMSE) - depression (CSDD) - mood (AMS) - function (FIM) - communication (CS) - behaviors (MBS)	Significant differences between experimental and control groups were found in 4 out of 6 measures: cognition, depression, positive mood and communication.	Medium
Kellett, Moyle, McAllister, King, Gallagher 2010 [52]Australia	Life stories and biography: a means of connecting family and staff to people with dementia.	To assess the Family Biography Workshop (FBW) designed to support family and staff to co- construct the history of the person with dementia in residential care.	A pilot study. Focus group interviews.	Qualitative	A purposeful sample of seven family careers and their relatives in care, seven care staff and one researcher in long term dementia care. Person with dementia: Gender: 1 woman and 6 men Relatives: Relationship to the person with dementia: 5 spouses and 2 children Staff: Profession: 8 other	The FBW process consisting of a series of six weekly two-hour sessions, involved families and staff completing a set of exercises designed to help them build a biography of the life of the person with dementia. Between the sessions, incrementally, both family members and staff shared biographical materials with the residents, and thus, involving the person with dementia. The FBW was used to provide a structured process and a defined role for family caregivers to assist staff in personalizing nursing care.	Family-staff caregiver - attitudes - perceptions of roles - conflict - subsequent management of stress	For family caregivers “standing outside” four characteristics: freeing the family from the present, breaking free of the disease-saturated narrative, gaining insight into grieving and healing and learning something about my relative not previously appreciated, were found. For staff “opening possibilities” four characteristics: having a point of reference to communicate with resident and family, appreciating the resident and family member as people bound by a family history, developing insight and understanding into behavior and developing confidence to relate as a person, were found. For person with dementia “knowing how” two characteristics shared by staff and family: knowing how to stimulate and provoke memories and knowing how to calm the person with dementia using biography, were found. “Complementing the organization” concerning organization issues, three characteristics: promoting community, the challenges of integrating FBW in practice and the complexities of division of labour and responsibility, were found.	Medium
McKeown, Clarke, Ingleton, Ryan, Repper 2010 [48] United Kingdom	The use of life story work with people with dementia to enhance person-centred care.	To explore the use of taking a Life Story Work (LSW) approach with people with dementia. To investigate the ways in which LSW: - is understood and developed in practice - is experienced by all participants - affects the delivery of care - affects outcomes of care	A multiple case study. Conversations, observations, interviews, and documentary analysis.	Qualitative	A purposeful sample of four people with dementia across four care settings for people with dementia, 12 multi-professional staff and three relatives. Person with dementia: Age: 80–88 years Gender: 2 women and 2 men Relatives: Gender: 3 women Relationship to person with dementia: 1 spouse and 2 children Staff: Profession: 1 RN and 11 other	A practice development approach with six key themes was applied to the LSW intervention. Participant were given the opportunity to develop the use of LSW in whichever way they choose; encouraging the use of creativity in practice. Three cases developed a life story book and one a pen picture.	Person-centered outcomes	Using LSW can enhance person-centred care. Three main themes: “from patient to person”, “can you hear me?” and “pride and enjoyment”, were found. For family careers: allowed them to uphold their relative’s personhood. For care staff: enabled them to see the person behind the patient. For the person with dementia: enabled their voice to be heard and enabled them to feel proud about themselves and their lives.	Medium
McKeown, Ryan, Ingleton, Clarke 2015 [49] United Kingdom	‘You have to be mindful of whose story it is’: the challenges of undertaking life story work with people with dementia and their family carers.	To critically appraise some of the challenges that may emerge through the process of undertaking Life story work (LSW). Recommend how such challenged may be overcome or minimized. Contribute to what is currently a gap in the LSW literature.	A multiple case study. Conversations, observations, interviews, and documentary analysis.	Qualitative	A sample of four people with dementia across four care settings for people with dementia, 12 multi-professional staff and three relatives. Person with dementia: Age: 80–88 years Gender: 2 women and 2 men Relatives: Gender: 3 women Relationship to person with dementia: 1 spouse and 2 children Staff: Profession: 1 RN and 11 other	A practice development approach with eight principles was applied to the life story work intervention. Three cases developed a life story book and one a pen picture.	Experiences of people with dementia, family careers and care staff in using life story work.	Several challenges may occur during the process of undertaking LSW. Four main themes “personal disclosures”, “whose story is it?”, “quality of life story books”, and “under and overuse of life story books”, were found.	Medium
Subramaniam,Woods, Whitaker 2014 [51] United Kingdom	Life review and life story books for people with mild to moderate dementia: a randomised controlled trial.	To evaluate the effect of different pathways for developing a life story book (LSB) for people with dementia.	A preliminary, randomized single blind controlled trial, with two parallel arms	Quantitative	A sequential individual-based randomization sample of 23 people with dementia (11 intervention, 12 control) living in 11 care homes participated, 23 relatives and 68 care staff. Person with dementia: Age: 73–99 years Gender: 16 women and 7 men Length of stay: 25–51 months CDR: Mild to moderate dementia Relatives: Age: 44–83 years Gender: 15 women and 8 men Relationship to person with dementia: 13 children and 10 other Staff: Age: 20–64 years Gender: 62 women and 6 men Profession: 3 RNs and 68 other Length of employment: 1–40 years	Life review /life story book intervention. Persons with dementia received 12 individual sessions undertaking the life review process leading to their own life storybook (LSB). Initially the focus is on childhood and adulthood, then family, home and adulthood. Finally there is a summary. Life story book as gift intervention. The researcher worked closely with the participant’s relative, meeting them 5–6 times over 12 weeks. Together they developed a life storybook illustrated with photos and pictures to be given as a gift for their relative who was not involved in the process.	The persons with dementia: Primary outcome: - quality of life (QOL-AD) Secondary outcome: - depression (GDS-12R) - autographical memory (AMIE) - relationship with their relatives (QCPR) Staff: - approaches to dementia (ADQ) - knowledge about residents (author developed questionnaire) Relatives: - relationship with the person with dementia (QCPR)	For persons with dementia: A significant between-group difference was found immediately after the life review session had been completed in favor of LSB group. No differences in quality of life between the LSB intervention and the LSB as gift intervention groups were found six weeks after receiving the LSB. For both groups QOL-AD had improved. At 12-week assessment, there was a significant improvement in scores for the LSB group compared with the LSB as a gift group. There was no significant differences in between the groups on depression. After twelve weeks relationship warmths, rated by the person with dementia, had improved in the LSB group. For staff: Six weeks after the LSB had been available, staff attitude had improved significantly. Staff knowledge regarding the residents improved significantly at twelve-week follow-up assessment. For relatives: Quality of relationship improved significantly after the LSBs were produced by either pathways.	High

### Methods of synthesis

An integrative analysis inspired by Sandelowski et al. [42] was conducted. The aim provided direction for the analyses of the results from the included papers.

## Results

The included studies represent research from 2006 to 2015 with three studies using quantitative design and two studies (presented in three papers) using qualitative design. Three of the papers are from the United Kingdom, one from the United States, one from Northern Ireland and one from Australia. The number of persons with dementia who participated was 70. In addition, there were 33 relatives and 159 members of staff. Five of the papers specified the age of the person with dementia, all papers specified gender, while three papers described the cognitive ability of the person with dementia. The relatives’ age and gender were specified in, respectively, one and three out of four papers, and their relationship to the person with dementia was described in all four papers. Among the five papers that included staff, four papers described the staff’s professions. The demographic characteristics of the participants included in the studies are shown in Table 1.

Results from the integrative analyses of the six papers show that the use of life stories might contribute to ‘Maintenance of the person with dementia as a whole person rather than a demented patient’ as it enabled the voice of the person with dementia to be heard, enabled relatives to see their near one as a whole person and enabled staff to understand persons with dementia and their relatives. However, several challenges in creating and using life stories in care were also revealed, as described later.

### Enable the voice of the person with dementia to be heard

The results showed that five of the papers concerned how the use of life stories might influence the person with dementia. The life story enabled the voice of persons with dementia to be heard and to feel proud about themselves and their lives [48]. However, enabling persons with dementia to tell their own life story could be a challenge due to memory loss. Joint authorship is referred to as a way of supporting the ability of persons with dementia to contribute to their own story [49].

After a life storybook process, persons with mild to moderate dementia scored significantly more positively on the outcome measures of cognition, depression, positive mood and communication than the control group [50]. Regarding quality of life as measured on the Quality of life-Alzheimer’s disease scale, to take part in the creation of their own life story book had significantly positive benefits for persons with dementia immediately after the life review session had been completed [51]. Their autobiographical memory also significantly improved. However, no difference was observed between the control and experimental group six weeks after having received the life storybook [51].

Kellett et al. [52] found that persons with dementia benefited when life stories were used, as both staff and relatives were more capable of stimulating and provoking memories, as well as knowing how to calm them when necessary.

### Enable relatives to see their near one as a whole person

The results showed that two of the papers described how the use of life stories might influence relatives. Family members appreciated that life story work made their near ones more visible, present and heard [48]. They could now focus on their near ones as persons who had lived a meaningful life and enjoyed accomplishments throughout their lifetime [52]. Reviewing memories released relatives from the immediate dementia-related care in their everyday experience, thus enabling them to see their near ones as a whole person in new and different ways – even in ways not previously appreciated [52].

The relatives enjoyed participating in the process of implementing life stories [48] and they experienced great comfort and confidence in observing the ability of the nursing staff to recognise, value and incorporate life stories in everyday care [52]. The relatives also gained insight into their own grieving with regard to the process of their near ones’ dementia illness, something that helped them to cope with it more positively [52].

### Enable staff to understand persons with dementia and their relatives

The results showed that five of the papers were about how the use of life stories might influence staff. Introducing a life story collage increased the staffs’ knowledge of the persons with dementia regarding family, jobs/careers, likes, dislikes and interests. It improved the relationship between staff and the persons with dementia, and the staffs’ involvement [53]. Life story work helped the staff to see the person with dementia as more than a patient and it enhanced their understanding of the person for whom they were caring [48]. The use of the family biography workshop, which is a structured process to facilitate the involvement of staff, family members and friends of the person with dementia in co-constructing biographies of their lives, gave the staff better knowledge of how to stimulate and provoke memories, and how to calm the person by using his/her biography. The staff discovered points of reference to communicate with the person with dementia and family and view them as part of a family history. The staffs’ understanding and insight into behaviour increased and they felt empowered to provide relationship-centred care rather than task-oriented care [52]. Further, staff attitudes to persons with dementia measured as hopefulness and person-centeredness, improved when a life story book was developed [51]. However, another study found that staff perceptions of individualised care or person-centred care practices did not improve significantly after introducing a life story collage [53].

McKeown et al. [49] found that life story work may be overused and underused, and that finding a balance is important. From the staff point of view it was sometimes a challenge when sensitive information and personal disclosures emerged uninvited. The results also showed that involving relatives could be a challenge as to whose story is uncovered. Questions were raised about the quality of the life story work when it had been created by relatives [49]. Developing a life story book either with the participation of the person with dementia or via relatives without involving the person with dementia improved the staff’s knowledge of the person with dementia regarding details such as hobbies, favourite food and school [51]. The process of creating life story work was perceived by staff as being enjoyable [48].

## Discussions

The results of this review show that research into the use of life stories’ and its influence on persons with dementia, their relatives and staff is still scarce. Even though the literature search resulted in 749 papers once the duplicates had been removed, it was found that the majority of studies focused on the importance of the phenomenon of life story work and, to a much lesser extent, on how to actually use it and its influence on persons with dementia, their relatives and staff. Furthermore, among the included studies, the quality of four of the studies was rated as medium quality and only one was rated as high quality.

The total sample covered in this review is small and the included studies provide little information about persons with dementia, their relatives and staff, as well as the overall context. No specific diagnoses of the persons with dementia were mentioned and only two of the studies provided information about the person’s MMSE score. Consequently, as little or nothing about the diagnoses and the extent of the person’s cognitive impairment are known, it is not possible to explore possible relationships between diagnoses, degree of cognitive impairment, the use of life story and its influence on the persons with dementia, their relatives and staff. This information could be of interest as previous research shows that the more severe the dementia symptoms, the less the care is individualised and the less the relatives are involved in the care [20].

The integrated and synthesised results from the quantitative and qualitative studies show that use of life stories might contribute to ‘Maintenance of the person with dementia as a whole person rather than a demented patient’. This result is in line with what is referred to as ‘seeing the person beyond the dementia’, which is one of the goals of dementia care [11, 20, 41]. As this group of persons are at risk of becoming objectified and seen as non-persons, especially as the disease progresses [11, 20], the use of life stories should be considered an important element of the care in order to preserve and enhance the dignity and well-being of the person with dementia. The latter has been highlighted as particularly important with regard to dementia care in several studies [20, 54, 55]. As no medical curative treatment of dementia exists [2, 7], individually tailored care is crucial in order to offer the increasing number of persons with dementia and their relatives [1] high-quality professional care in the future.

As the results revealed several challenges both in creating and using the life stories in care, life story work has to be taken into consideration with a high level of sensitivity by the staff. It is therefore both worrying and striking that among the staff, there were only a few RNs. This might be a coincidence, but most likely is not. According to previous research, nursing homes are facing major challenges regarding a serious lack of personnel with formal health education [56] and the utmost need for a high degree of expertise in order to provide high-quality care is emphasised [30].

### Methodological considerations

Using an integrated review method provides an opportunity to present a comprehensive understanding of a phenomenon of relevance to health care [57]. Even though only three quantitative and two qualitative studies were included in this review, presenting the existing knowledge is valuable, in order to start creating a knowledge base for using the persons’ life stories in dementia care, and also to uncover areas for further research.

The four researchers worked systematically in pairs to minimize subjectivity. The quality of the papers was assessed using a reliable quality assessment tool [45]. Any uncertainties were discussed in the research team until consensus was reached. This increased the validity and reliability of the selection and quality assessment process [43, 57].

Different concepts have been used for describing the use of life stories, which made the search process challenging. To strengthen the validity, we elected to use five concepts based on our own knowledge of the field. These concepts were life histories, life story, narratives, diary and biographical approach. No further concepts were identified during the search process. To strengthen the reliability, a search update was performed in September 2015. No further studies were found. References in the chosen papers were scrutinised, but searches for ‘grey literature’ were not conducted.

## Conclusions

The use of life stories was generally found to have a positive influence on persons with dementia, their relatives and staff. The theme ‘Maintenance of the person with dementia as a whole person rather than a demented patient’, was identified as representing a positive influence. Nevertheless, the total sample of studies was few, participants in the included studies were limited and the quality of four of the studies was assessed as medium. This result indicates that a rather simple intervention might have the potential to make positive changes for persons with dementia, their relatives and staff. There is a huge need for studies, including randomised controlled trials, to measure the impact of the use of life stories on physiological and psychological aspects of persons with dementia, and also how it influences their relatives and staff.
